# Supplementary material for: Evaluation of Increasing Dietary Concentrations of a Multi-Enzyme Complex in Feedlot Lambs’ Rations
Source: Animals (Basel). 2024 Apr 18;14(8):1215. doi: 10.3390/ani14081215 (PMC11047411; doi:10.3390/ani14081215)
Supplement: Supplementary file 1 [file animals-14-01215-s001.zip › animals-2949352-supplementary.pdf]

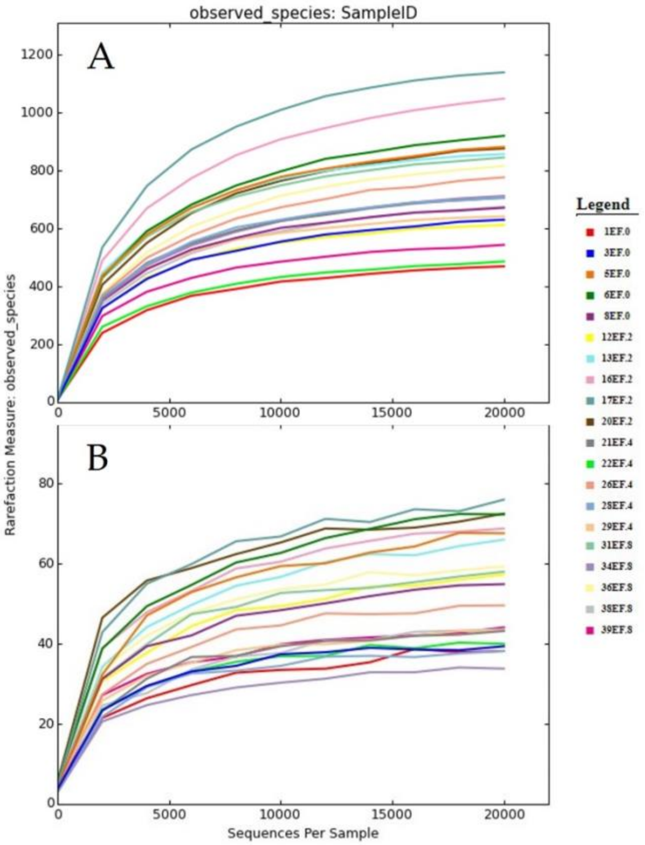

Figure S1. Rarefaction curve of observed species number at ASV-level of (A) bacterial/ archaeal and (B) eukaryotic (fungi/protozoan) diversity
